# Integrated proteomics and phosphoproteomics revealed druggable kinases in neoadjuvant chemotherapy resistant tongue cancer

**DOI:** 10.3389/fcell.2022.957983

**Published:** 2022-10-28

**Authors:** Irene A. George, Gajanan Sathe, Vivek Ghose, Anuradha Chougule, Pratik Chandrani, Vijay Patil, Vanita Noronha, R. Venkataramanan, Sewanti Limaye, Akhilesh Pandey, Kumar Prabhash, Prashant Kumar

**Affiliations:** ^1^ Institute of Bioinformatics, Bangalore, India; ^2^ Manipal Academy of Higher Education (MAHE), Manipal, India; ^3^ Medical Research Council (MRC) Protein Phosphorylation and Ubiquitylation Unit, School of Life Sciences, University of Dundee, Dundee, United Kingdom; ^4^ Tata Memorial Hospital (TMH), Mumbai, India; ^5^ Karkinos Healthcare Pvt Ltd., Mumbai, India; ^6^ Sir H.N. Reliance Foundation Hospital and Research Centre, Mumbai, India; ^7^ Center for Molecular Medicine, National Institute of Mental Health and Neurosciences (NIMHANS), Bangalore, India; ^8^ Department of Laboratory Medicine and Pathology, Centre for Individualized Medicine, Mayo Clinic, Rochester, MN, United States

**Keywords:** tongue cancer, proteome, phosphoproteome, neoadjuvant chemotherapy resistance, rho GTPases signaling

## Abstract

Tongue squamous cell carcinoma is an aggressive oral cancer with a high incidence of metastasis and poor prognosis. Most of the oral cavity cancer patients present in clinics with locally advanced unresectable tumors. Neoadjuvant treatment is beneficial for these individuals as it reduces the tumor size aiding complete resection. However, patients develop therapy resistance to the drug regimen. In this study, we explored the differential expression of proteins and altered phosphorylation in the neoadjuvant chemotherapy resistant tongue cancer patients. We integrated the proteomic and phosphoproteomic profiles of resistant (n = 4) and sensitive cohorts (n = 4) and demonstrated the differential expression and phosphorylation of proteins in the primary tissue of the respective subject groups. We observed differential and extensive phosphorylation of keratins such as KRT10 and KRT1 between the two cohorts. Furthermore, our study revealed a kinase signature associated with neoadjuvant chemotherapy resistance. Kinases such as MAPK1, AKT1, and MAPK3 are predicted to regulate the resistance in non-responders. Pathway analysis showed enrichment of Rho GTPase signaling and hyperphosphosphorylation of proteins involved in cell motility, invasion, and drug resistance. Targeting the kinases could help with the clinical management of neoadjuvant chemotherapy-resistant tongue cancer.

## 1 Introduction

Oral cavity cancer (OCC), one of the prevalent cancers worldwide, is a subgroup of head and neck cancers. Every year 600,000 individuals are diagnosed with head and neck cancers which include the oral cavity, pharyngeal and larynx cancers [International Head and Neck Cancer Epidemiology Consortium] (International Head and Neck Cancer Epidemiology Consortium, 2017). OCC ranks among the top three types of cancers in India with high incidence and mortality in males (GLOBOCAN, 2020) (Sharma et al., 2018). OCC occurs on the lining of the lips, mouth, or upper throat. The major etiologic factors include cigarette smoking, alcohol intake, and infection with human papilloma virus (Haddad and Shin, 2008).

Tongue squamous cell carcinoma (TSCC), the most common and aggressive oral cancer (OC) is characterized by rapid progression, metastasis, and poor prognosis (Elango et al., 2011). The incidence of TSCC in India is second in the world and is more common among young adults (Mathew Iype et al., 2001). The conventional treatment for OCC includes chemotherapy, radiotherapy, and surgery. Taxanes, anthracyclines, platinums, and antimetabolites are the commonly used chemotherapeutic drugs in OCC. The treatment selection of these patients will depend upon the stage of disease progression. For stages I and II, the removal of the tumor through surgery or radiation therapy is suggested; for late-stage cancers, chemotherapy, radiotherapy, or combination therapies along with surgery will be carried out. The conventional therapeutic methods are restricted by the development of resistance, disfiguring after surgery, and functional side effects (Furness et al., 2011). Recent studies have shown neoadjuvant chemotherapy (NACT) as a beneficial strategy, as it reduces the positive margins, deformity, functional morbidity, and locoregional control of locally advanced technically unresectable tumors (Patil et al., 2014; Vishak et al., 2015). NACT is applicable for technically unresectable patients with locally advanced OCC (Jain and Pounikar, 2018). In these patients, docetaxel 75 mg/m2 on D1, cisplatin 75 mg/m2 on D1 with 5FU, 750 mg/m^2^ D1-4 are administered every 3 weeks (DCF). DCF is considered as the standard chemotherapy agent for patients with locally advanced head and neck squamous cell carcinoma (Roy and Chatterjee, 2013). Nevertheless, significant low response or local relapses have also been reported in NACT-treated patients (Joshi et al., 2013; Yanamoto et al., 2014). For example, a retrospective analysis in Indian cohort with technically unresectable oral cancer treated with combinations of a taxane and a platinum with or without 5-fluorouracil showed an overall response rate of only 25.1% (Patil et al., 2014). Chemoradiation therapy is also a treatment choice for the patients with technically unresectable tumors. However, compared to patients undergoing chemoradiation, patients who underwent surgical treatment followed by postoperative adjuvant treatment had better loco control of their disease and overall survival (Bernier et al., 2004; Bernier et al., 2005; Patil et al., 2014; Fagan et al., 2021). Therefore, it is very clinically beneficial to comprehend the molecular mechanism involved in NACT treatment resistance.

Oral cavity cancer patients show differential response to first-line chemotherapies such as 5-fluorouracil (5-FU), cisplatin, and docetaxel, as well as targeted drugs. Recently, some studies have explored the mechanisms underlying resistance in tongue cancer. Peng *et al.*, has reported increased expression of tongue cancer chemotherapy resistance-associated protein1 (TCRP1) result in cisplatin resistance (Peng et al., 2012). Independent studies showed the association of zinc finger E-box-binding homeobox 1 (ZEB1), c-Myc, MACC1, and GSK3β with cisplatin resistance (Han et al., 2018). Although independent studies reported the association of molecules with resistance, the complete proteomic landscape of treatment resistance in tongue cancer is not yet explored. In this study, we aim to identify differentially expressed proteins and aberrantly activated kinases involved in neoadjuvant chemotherapy resistance.

In this study, we employed liquid chromatography-tandem mass spectrometry (LC-MS/MS) to profile the proteome and phosphoproteome of NACT resistant tongue cancer patients. Recently, mass spectrometry (MS)-based proteomics study has been widely employed in cancer research as it ensures high throughput identification and quantification of differentially expressed proteins and kinases involved in disease onset, progression, and prognosis (Deb et al., 2019; Puttamallesh et al., 2020; Sathe et al., 2020; Kumar et al., 2021). The application of a quantitative proteomic approach enabled us to identify the differentially expressed proteins in NACT resistant and sensitive cohorts. Further, we employed TMT labeling along with IMAC based phosphopeptide enrichment to identify enriched kinases and activated pathways involved in NACT resistance. The quantitative proteomic and phosphoproteomic analysis identified dysregulation in cytoskeletal associated proteins, and enrichment of Rho GTPase signaling pathways in NACT resistant tongue cancer patients. To our knowledge, this is the first study that profiled the proteome and phosphoproteome of NACT resistant tongue cancer patients. Our data provide an insight into the molecular mechanisms of NACT resistance which could help to circumvent treatment resistance in the future.

## 2 Materials and methods

### 2.1 Patient sample collection

**FIGURE 1:**
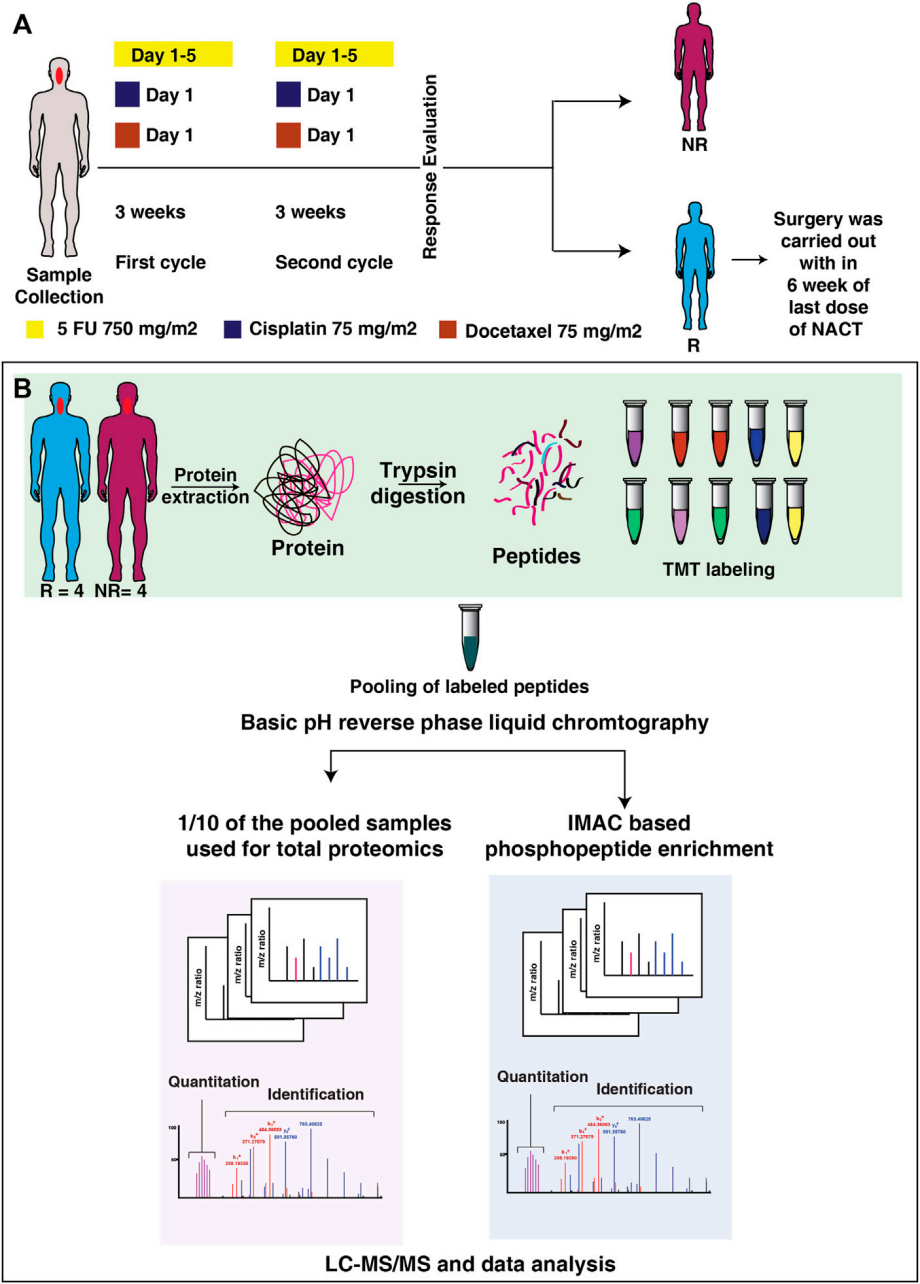
Treatment timeline and the workflow applied in integrated proteomic and phosphoproteomics of NACT resistant and sensitive TSCC patients. **(A)** The timeline of the tissue collection and treatment given to the patient. Tissue biopsy samples were collected from the patient prior to the start of therapy. All the patients were treated with DCF, or DC based neoadjuvant chemotherapy. After two cycle of treatment patients were classified as resistant or sensitive according to RECIST criteria. **(B)** Workflow applied for integrated proteomic and phosphoproteomics of NACT resistant and sensitive tongue cancer. Tissue biopsy from treatment naïve patients were homogenized to extract proteins. The extracted protein was digested with trypsin and each sample was labelled with 10-plex Tandem Mass Tags (TMT) labelling kit. Following to labelling samples were pooled and one-tenth of the pooled sample was used for global proteomics experiment and remaining sample was enriched for phosphoproteomic experiment. Samples were then analyzed in Orbitrap-Fusion-Tribrid mass spectrometer. The raw files were then searched against Mascot and Sequest HT search engines, followed by data analysis in Perseus. Exe 1.6.5.0.

The tissue biopsy samples from treatment-naive tongue squamous cell carcinoma patients were collected from the patients enrolled in Tata Memorial Hospital, Mumbai, India and the tumor content of the samples was verified by a pathologist. The study was approved by the institutional ethical committee and a consent waiver was given. All patients were undergone neoadjuvant chemotherapy with standard dosages. The patients were administrated with DCF- Docetaxel 75 mg/m2 on day 1, Cisplatin 75 mg/m2 on day 1 and 5 FU 750 mg/m2 on Day 1 to five or DC- Docetaxel 75 mg/m2 on day 1, Cisplatin 75 mg/m2 on day 1. Both regimens were given for 3 weeks duration. The response of the patients towards the treatment was recorded after the second cycle (for one patient treatment evaluation was carried out after first cycle) of treatment. The response evaluation of the patients was analyzed using RECIST (Response Evaluation Criteria in Solid Tumors) (Eisenhauer et al., 2009). Patients were classified as responders or non-responders based on the tumor size reduction or progression of disease respectively. The tumor size assessment was done using MRI scan. Patients with regressed tumor (responders) underwent surgery within 6 weeks of the last dose of NACT. The clinicopathological details of the patients are included in Supplementary Table S1. The treatment timeline is depicted in Figure 1A. The tissue samples were stored at -80°C until use.

### 2.2 Protein digestion and TMT peptide labeling

Protein was extracted and estimated using BCA (Bicinchoninic Acid) protein assay (Pierce, ThermoFisher Scientific). Reduction, alkylation, and precipitation of an equal amount of protein from each sample were performed with 100 mM (dithiothreitol) DTT, 20 mM iodoacetamide (IAA), and ice-cold acetone. The samples were then digested with trypsin (1:20, Promega) 12–16 h at 37°C and the peptides were cleaned using Sep-Pak C18 Plus Light cartridge (Waters, Catalog # WAT023501). Peptides were labeled with 10 plex TMT (Tandem Mass Tags) labels (ThermoFisher Scientific) according to the manufacturer’s protocol and the reaction was quenched by the addition of 5% hydroxylamine. The workflow of the experiment is depicted in Figure 1B **.**

### 2.3 Basic pH RPLC (bRPLC) and IMAC-based phosphopeptide enrichment

The pooled peptides were fractionated by basic pH liquid chromatography analyzed on Orbitrap-Fusion-Tribrid mass spectrometer (ThermoFisher Scientific), 10% of each fraction were used for global proteome and the remaining 90% of the peptides from each fraction were subjected to IMAC based phosphopeptide enrichment. The enriched phosphopeptides were eluted with 40 µl of 50% of ACN and 0.1% TFA. The peptides were resuspended in 30 µl of 0.1% TFA and desalted using C18 Stage Tips (Thermo Fisher Scientific). LC-MS/MS analysis of the eluted peptides was performed.

### 2.4 LC-MS/MS analysis

The peptides and enriched phosphopeptide analyses were carried out on an Orbitrap Fusion Tribrid mass spectrometer (Thermo Fisher Scientific) interfaced with an Easy-nLC II nanoflow liquid chromatography system (Thermo Fisher Scientific). Each fraction was then reconstituted in Solvent A (0.1% formic acid) and loaded on a trap column (75 μm × 2 cm) packed with Magic C18 AQ (Michrom Bioresources, Inc. Auburn, CA, United States). Peptides were resolved on an analytical column (75 μm × 15 cm) at a flow rate of 350 nL/min using a linear gradient from 5% to 60% ACN in a 120 min run. The following parameters were used for MS data acquisition: scan range of 400–1,600 m/z at a mass resolution of 120,000, and the MS/MS data were acquired with a resolution of 30,000 at m/z of 400. The data-dependent acquisition was carried out where the most intense precursor ions were detected.

### 2.5 Data analysis

The data were then searched against the Human RefSeq protein database (version 81, containing protein entries with common contaminants) using the SEQUEST search algorithm through the Proteome Discoverer platform (version 2.1, Thermo Scientific). Search parameters for Proteome Discoverer analysis were as follows: two missed cleavages allowed, trypsin as cleavage enzyme, a tolerance of 10 ppm on precursors, and 0.02 Da on the fragment ions. The fixed modification includes carbamidomethylation at cysteine, TMT 10-plex (+229.163) modification at N-terminus of peptide and lysine, and variable modification as oxidation of methionine and deamidation of asparagine and glutamine. For the phosphoproteomic analysis, oxidation of methionine; the phosphorylations of serine, threonine, and tyrosine; and the deamidation of asparagine and glutamine were selected as dynamic modifications. Data were also searched against a decoy database and filtered with a 1% false discovery rate (FDR). The mass spectrometry proteomics data have been deposited to the ProteomeXchange Consortium *via* the PRIDE partner repository with the dataset identifier PXD037356.

For comparing our patient data with publicly available databases we used cBioPortal (https://www.cbioportal.org/). The mRNA and protein expression of the predicted kinases were checked using the study Head and Neck Squamous Cell Carcinoma (TCGA, Firehose Legacy) and RPPA (Reverse phase protein arrays in signaling pathways) data respectively.

### 2.6 Statistical analysis

For the identification of differentially expressed proteins and differentially phosphorylated proteins between responders and non-responders, two samples “*t*-test” (*p* < 0.05) were used. Fold change of proteins and phosphopeptides were calculated by taking the ratio of average expression or phosphorylation of proteins in non-responders and responders (fold change cut off <1.5).

### 2.7 Clustering analysis

Unsupervised clustering of proteome and phosphoproteome datasets was conducted separately using the online tool Morpheus (Broad Institute https://software.broadinstitute.org/morpheus/). Normalized abundance values of the significantly expressed proteins and significantly phosphorylated proteins were used for clustering analysis.

### 2.8 Pathway analysis and protein-protein interaction network

Reactome analysis tool (http://www.reactome.org/) was used to identify the enriched pathways using the set of differentially expressed genes (*p* ≤ 0.05) (https://www.reactome.org/PathwayBrowser/#TOOL = AT).

Protein-protein interaction network was generated using STRING database. The proteins which are dysregulated only at the phosphorylation level were using for constructing the interaction network (https://string-db.org/).

### 2.9 Kinome map and kinase enrichment analysis

The list of identified kinases was searched and a kinome map was constructed using the KinMap tool (http://www.kinhub.org/kinmap/index.html). Dysregulated kinases (hyper- or hypo-phosphorylated) were highlighted in the KinMap. Kinase-substrate enrichment analysis was carried out using Kinase Enrichment Analysis 3 (KEA3) (https://maayanlab.cloud/kea3/). The hyperphosphorylated and hypophosphorylated proteins were given as input to predict the enriched kinases in the data set. The upstream phosphatases for the downregulated kinases were identified using PhosphoSitePlus (www.phosphosite.org).

## 3 Results

### 3.1 Quantitative analysis of proteome and phosphoproteome of NACT resistant TC patients

To assess the protein expression and phosphorylation events involved in treatment resistance, we quantified the proteome and phosphoproteome of NACT resistant (n = 4) and sensitive (n = 4) tongue cancer patients using multiplexed TMT and LC-MS/MS approaches. We collected the tissue from treatment-naive tongue squamous cell carcinoma patients and the response towards the treatment was recorded according to RECIST criteria. The samples were digested with trypsin followed by labeling with 10 plex TMT tags and pooling. The pooled sample was fractionated by basic pH liquid chromatography. 10% of each fraction was used for global proteome and the remaining 90% of the peptides from each fraction were subjected to IMAC based phosphopeptide enrichment. The MS data were processed and searched against databases using SEQUEST-HT and MASCOT algorithms using the Proteome Discoverer 2.0 platform. The workflow of the experiment is depicted in Figure 1B.

**FIGURE 2:**
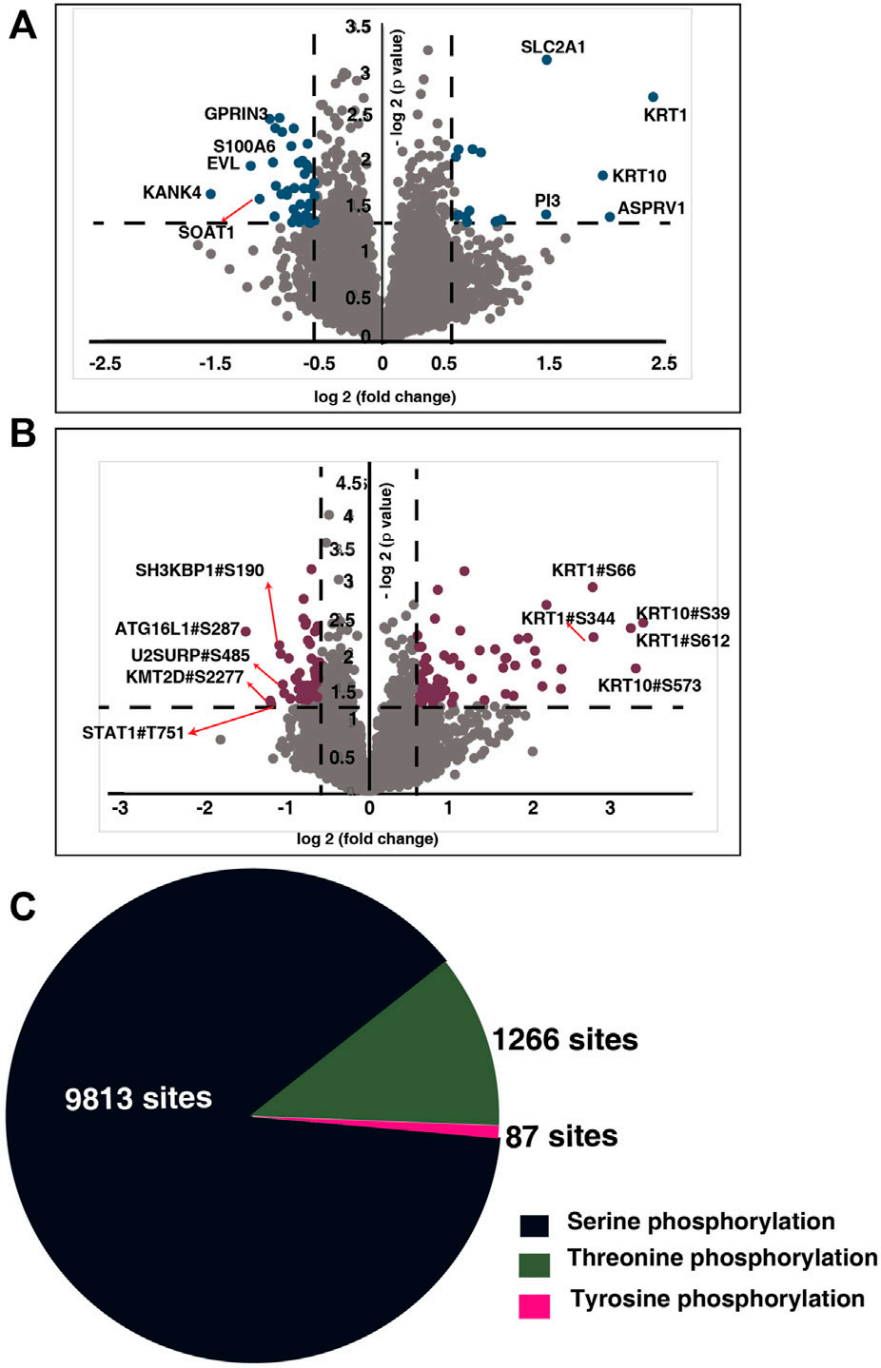
Global proteome and phosphoproteome of NACT resistant and sensitive TSCC patients. **(A)** Volcano plot of global proteome of NACT resistant and sensitive tongue cancer patients. Fold change of the protein abundances (log2) are plotted against the *t*-test *p*-values (-log10). Dashed lines represent significant levels. Data points in blue color are significantly dysregulated proteins (fold change cut off>1; *p*-value<0.05). **(B)** Volcano plot of phosphoproteome of NACT resistant and sensitive tongue cancer patients. Fold change of the protein abundances (log2) are plotted against the *t*-test *p*-values (-log10). Dashed lines represent significant levels. Data points in maroon color are significantly phosphorylated proteins (fold change cut off>1; *p*-value<0.05). **(C)** Pie chart representing the number of serine, threonine and tyrosine phosphosites identified in our study.

We compared the global proteome and phosphoproteome of the NACT resistant patients with the sensitive cohorts. From our global proteome data, we identified 7,453 proteins and quantified 7,000 proteins present across all the samples **(** Figure 2A) (Supplementary Tables S2, S3). To identify the probability of Serine/Threonine/Tyrosine phosphorylation site we employed phosphoRS algorithm with a cut-off of >75% (Patil et al., 2018). We identified 15,440 phosphopeptides corresponding to 4,150 proteins (Supplementary Table S4). We quantified 9,385 phosphopeptides corresponding to 3,106 proteins across all the patients (Figure 2B) (Supplementary Table S5). Of the quantified phosphosites, 9,813 are serine, 1,266 are threonine, and 87 belong to tyrosine phosphorylation (Figure 2C).

### 3.2 Altered proteome and phosphoproteome of NACT resistant TSCC patients

**FIGURE 3:**
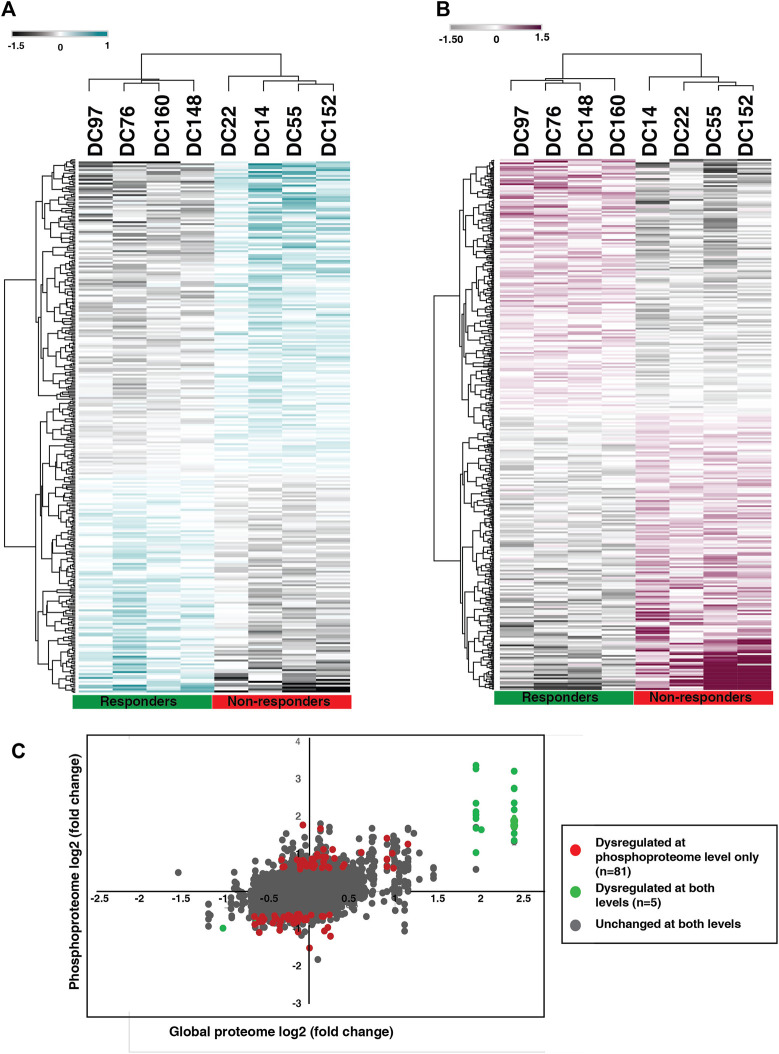
Proteomic and phosphoproteomic signature of NACT resistant TSCC patients. **(A)** Unsupervised clustering of significantly expressed proteins between NACT resistant and sensitive patients. Abundance values of significantly expressed proteins are represented in the heatmap. **(B)** Unsupervised clustering of significantly phosphorylated peptides in NACT resistant and sensitive patients. Abundance values of significantly phosphorylated peptides are represented in the heatmap. **(C)** Scatter plot comparing the protein expression and phosphorylation. Red datapoints represent differential phosphorylation with fold change ≥2 without any change in proteomic data and green datapoints with fold change ≥2 that are changed in both proteomic as well as phosphoproteomic data. Grey datapoints represent proteins that are unchanged in both datasets.

To identify the altered protein expression and phosphorylation between the NACT resistant and sensitive cohort we carried out statistical analysis for the proteomic as well as phosphoproteomic data separately. Our proteomic data analysis identified significant expression of 275 proteins of which 59 proteins are differentially expressed with a fold change cut-off of 1.5 **(** Supplementary Table S6 **).** Unsupervised clustering of the significantly expressed proteins revealed distinct proteomic signatures in the resistant cohort (Figure 3A). Furthermore, from the phosphoproteomic data, we identified significant phosphorylation of 305 phosphopeptides corresponding to 254 proteins that clustered based on their response towards the treatment (Figure 3B) (Supplementary Table S7). We observed differential phosphorylation of 126 phosphopeptides corresponding to 98 proteins (fold change> 1.5). In the proteomic data, we observed high expression of proteins such as keratin 1(KRT1) (fold change = 5.31), aspartic peptidase retroviral like 1 (ASPRV1) (fold change = 4.06), keratin 10 (KRT10) (fold change = 3.89), solute carrier family two member 1(SLC2A1) and peptidase inhibitor 3 (fold change = 2.73) in non-responders. Further, in our phosphoproteomics data, we observed extensive and high phosphorylation of different keratins. More than five sites of KRT10 and KRT1 were hyperphosphorylated in non-responders. Hyperphosphorylation of KRT10 at the sites S39, S537, S577, S42, S459, S56, S61, and S33; KRT1 at the sites S612, S344, S66, Y639, S13, S609, S618, and S541 were observed. We also compared between the differentially expressed proteins and differentially phosphorylated proteins. We observed 81 proteins were dysregulated at phosphorylation level without any change in protein expression and five proteins showed significant changes at both proteomic as well as phosphoproteomic levels (Figure 3C). Protein-protein interaction network with the 81 proteins was constructed (Supplementary Figure S1, Supplementary Table S8).

### 3.3 Functional enrichment of differentially expressed proteins and phosphorylated proteins

**FIGURE 4:**
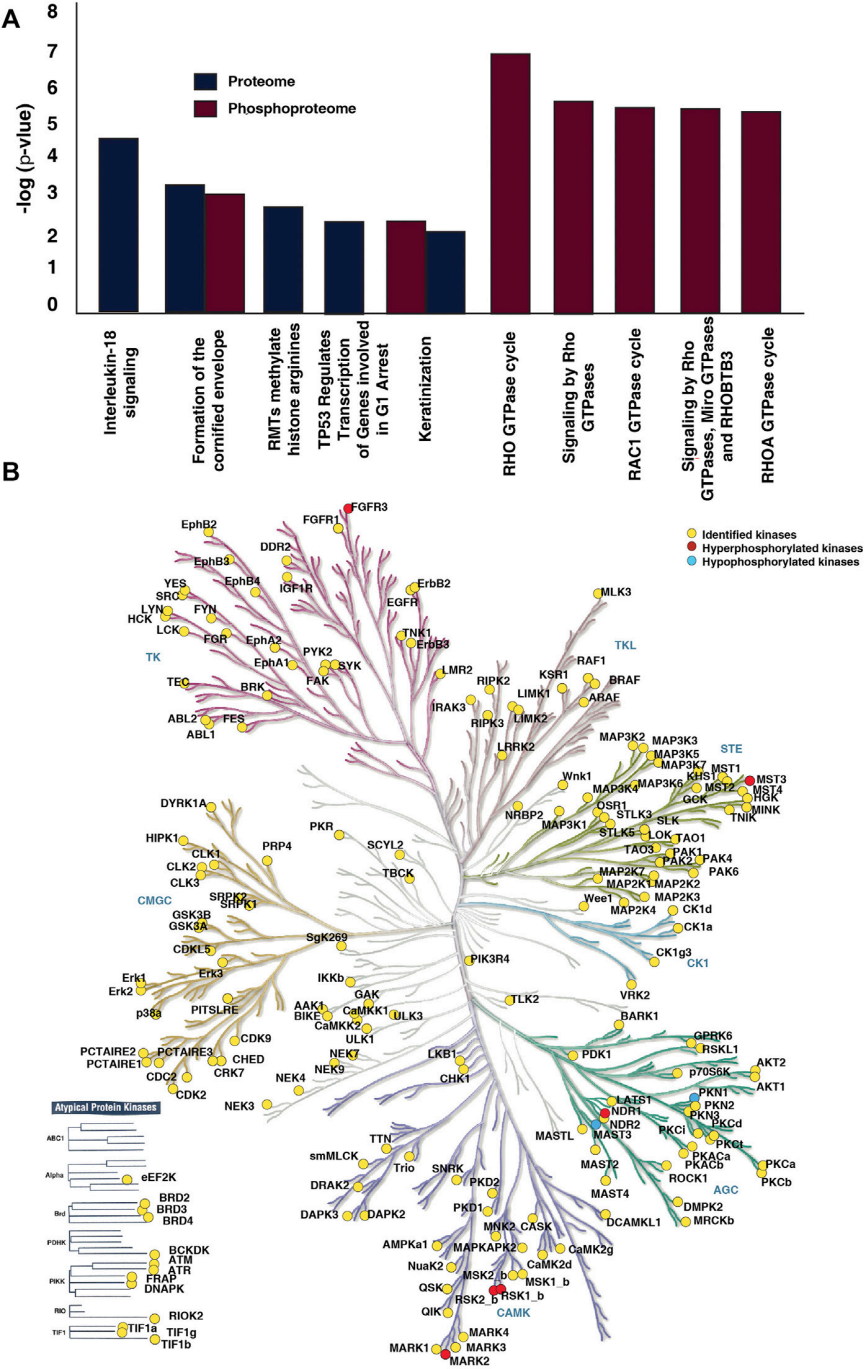
Pathways and kinases enriched in NACT resistant TSCC patients. **(A)** Top ten pathways significantly enriched in proteomic and phosphoproteomic data. Reactome pathway analysis of dysregulated proteins and differentially phosphorylated proteins revealed dysregulation of many pathways. Formation of cornified envelope is the most enriched in proteomic data. In phosphoproteomic data Rho GTPase cycle, Signalling by Rho GTPases, Signaling by Rho GTPases, Miro GTPases and RHOBTB3 were top three enriched pathways. **(B)** Kinases identified in the dataset. The kinome map was generated using KinMap tool.

Functional analysis of differentially expressed proteins and differentially phosphorylated proteins revealed enrichment of common and distinct pathways. Pathway analysis of differentially expressed proteins revealed interleukin-18 signaling, formation of the cornified envelope, RMTs methylate histone arginines and TP53 Regulates Transcription of Genes Involved in G1 Cell Cycle Arrest and keratinization as top five dysregulated pathways (Supplementary Table S9). Whereas, the differentially phosphorylated proteins showed prominent enrichment of pathways associated with the Rho GTPase cycle and its signaling (Supplementary Table S10) (Figure 4A). 17 proteins involved in the Rho GTPase cycle are differentially phosphorylated in the non-responder cohort. We observed hyperphosphorylation of proteins such as Macoilin (MACO1) (S332; fold change = 1.77), kinectin (KTN1) (S110; fold change = 1.80), ADP-ribosylation factor GTPase-activating protein 2 (ARFGAP2) (S407; fold change = 1.82), Desmoglein (DSG) (S1031; fold change = 2.06), CDC interacting protein-4 (TRIP10) (S495; fold change = 1.71) which are involved in the Rho GTPase signalling pathway. The pathways such as the formation of cornified envelope and keratinization were enriched in both proteomic and phosphoproteomic data.

### 3.4 Altered kinases identified from the phosphoproteomics data

We further investigated the kinases involved in the differential phosphorylation events in the patients. We identified 191 kinases in our data set (Figure 4B). Thirteen of the kinases were atypical kinases, while 29 belonged to the tyrosine kinase (TK) family, 11 to the tyrosine like kinase (TLK) family, 34 to the homologs of yeast Sterile 7, Sterile 11, and Sterile 20 kinases (STE) family, 29 to the A, G, and C families (AGC) family, 42 to the calmodulin/calcium-regulated kinase (CAMK) family. We observed hyperphosphorylation of kinases such as Serine/threonine-protein kinase 3 (STK3/NDR1), Microtubule affinity-regulating kinase 2 (MARK2), mammalian Ste20-like protein kinase 3 (MST3), Fibroblast growth factor receptor 3 (FGFR3), Ribosomal protein S6 kinase alpha-1 (RPS6KA1) and Ribosomal protein S6 kinase alpha-3 (RPS6KA3) in non-responder cohort.

### 3.5 Predicted kinases activated in the NACT resistant TSCC patients

**FIGURE 5:**
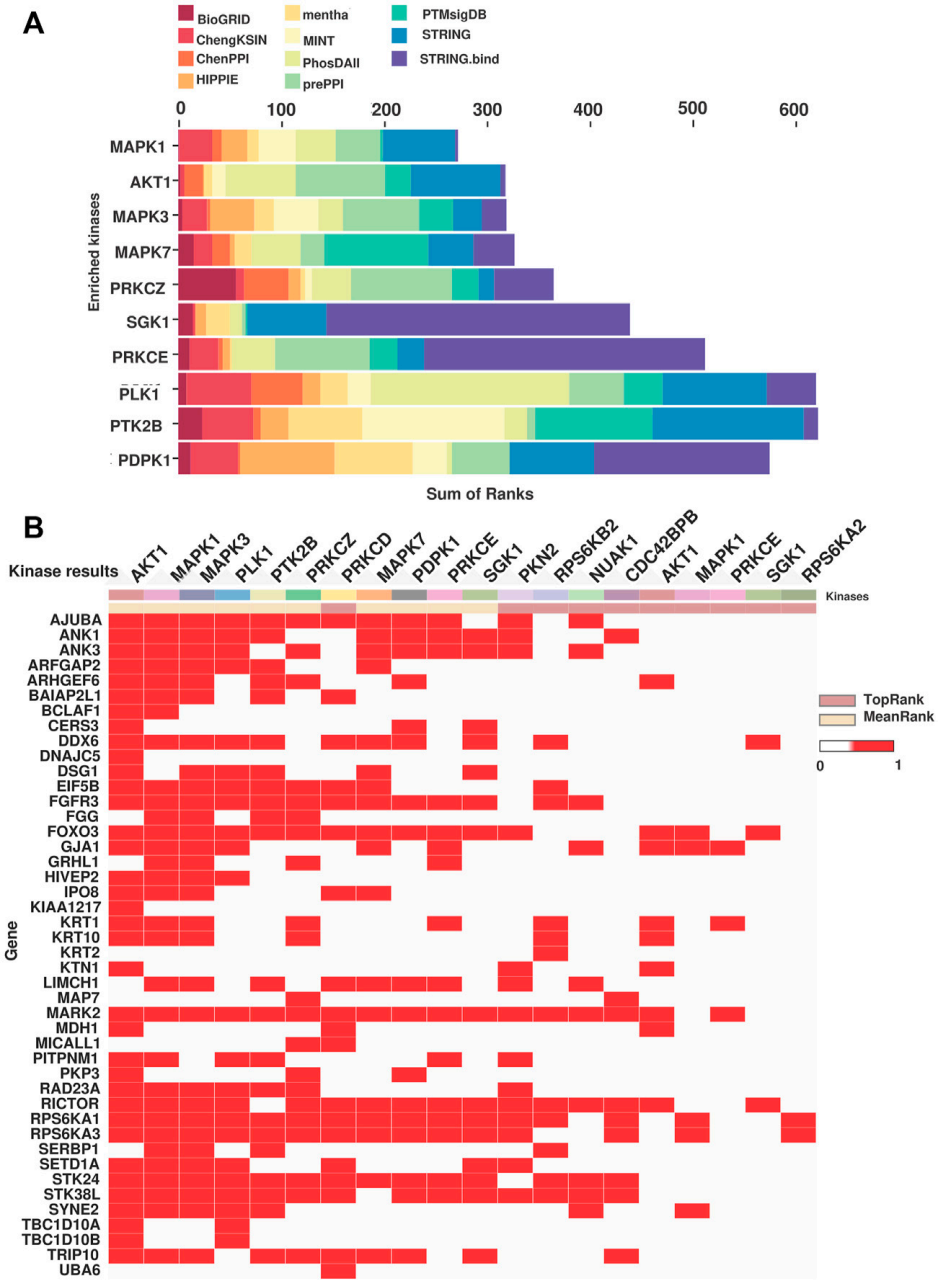
Kinase predicted to be enriched in the NACT resistant TSCC patients. **(A)** Bar chart representing the MeanRank visualization from KEA3 for the hyperphosphorylated proteins. Top ten kinases are plotted against the integrated ranking of the predicted kinases across different libraries based on MeanRank score. The bar is color coded by the library. **(B)** The clustergrammer visualization representing the top 10 ranked kinases based on the MeanRank and TopRank methods and putative substrates of the kinases. Each row are putative substrates from the input list and the column represent top 10 MeanRank and TopRank kinases.

To identify the molecular regulatory mechanism involved in treatment resistance in tongue cancer patients, we carried out kinase enrichment analysis. The top ten kinases enriched for the hyperphosphorylated proteins are depicted in Figure 5A. Mitogen-activated protein kinase 1 (MAPK1), RAC-alpha serine/threonine-protein kinase (AKT1), Mitogen-activated protein kinase 3 (MAPK3), Mitogen-activated protein kinase 7 (MAPK7), and Protein kinase C, zeta (PKCζ) are the top five ranked enriched kinases. We observed hyperphosphorylation of the substrates of these kinases in our data set. Figure 5B represents the clustergrammer visualization of the substrates hyperphosphorylated in the dataset and the predicted kinases. For example, the substrates of MAPK3 such as RPS6KA1 (fold change = 1.90), FGFR3 (fold change = 2.25), STK38 L (fold change = 1.82), MARK2 (fold change = 3.42), DSG1 (fold change = 2.06) were hyperphosphorylated. Multiple kinases upstream of the top hyperphosphorylated proteins such as KRT1, KRT10, MARK2, and FGFR3 were predicted to be enriched. The ranking, mean rank, and the substrates of the enriched top ten kinases are listed in Supplementary Table S11.

The top two kinases enriched for the hypophosphorylated proteins are SRC and ABL1, implying low activity in non-responders (Supplementary Figure S2). The phosphatases upstream of the top ranked downregulated kinase of non-responders were further identified from PhosphoSitePlus. PPP2CA, PTEN, PTP1B, PTPN13, PTPRA, PTPRJ, and SHP-2 are the upstream phosphatases identified for the kinase SRC.

### 3.6 mRNA and protein expression of the predicted kinases in tongue cancer patients

**FIGURE 6:**
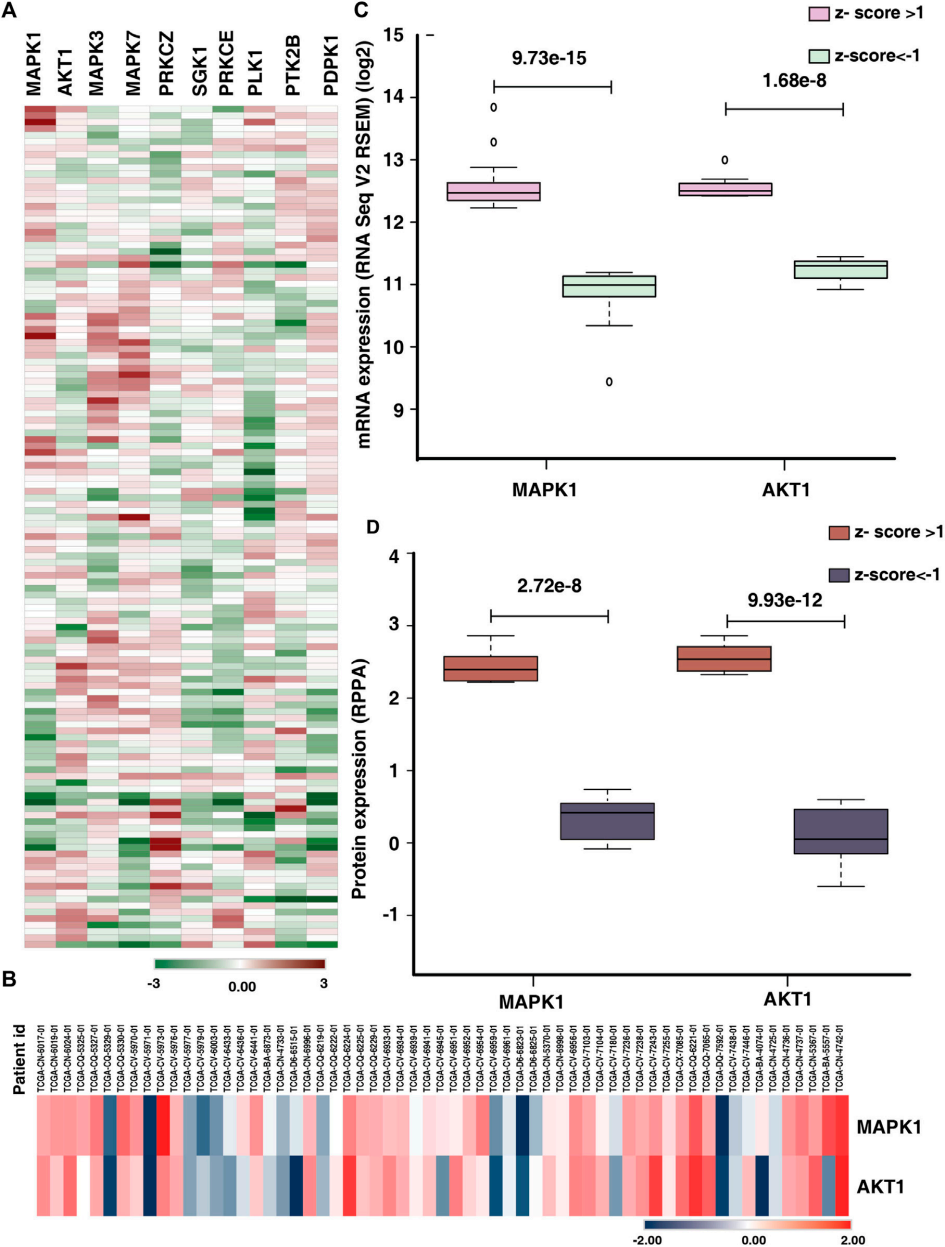
Comparison of expression of predicted kinases in publicly available data. **(A)** Heat map representing the mRNA expression (z-score) of top ten predicted kinases in tongue cancer patients (131 patients) in the study Head and Neck Squamous Cell Carcinoma (TCGA, Firehose Legacy). **(B)** Protein expression (z-score) of the kinases MAPK1 and AKT1 of tongue cancer patients (RPPA data). **(C)** Comparison of the mRNA expression of top two predicted kinases (MAPK1 and AKT1) between the upregulated (z-score>1) and downregulated groups (z-score < -1). **(D)** Box plot representing the significant difference in protein expression of top two predicted kinases (MAPK1 and AKT1) in upregulated group (z-score>1) and down regulated group (z-score < -1).

The mRNA and protein expression of the top ten predicted kinases were checked in the publicly available data sets using cBioPortal. For our analysis, we used the study Head and Neck Squamous Cell Carcinoma (TCGA, Firehose Legacy), which included 133 individuals with tongue cancer. In the 131 patients for whom expression data were available, we examined the mRNA expression of the predicted kinases identified in our study. In the TCGA data also we observed variable expression of the predicted kinases across the tongue cancer patients (Figure 6A). Based on the TCGA data, 30%, 21%, 31%, 26%, and 22% of the patients, respectively, have altered expression of MAPK1, AKT1, MAPK3, MAPK7, and PRKCZ (z-score>1) (Supplementary Figure S1). We further checked the protein expression of the predicted kinases in the RPPA data using cBioPortal. In RPPA, protein expression of 61 tongue cancer patients were available and the expression of only the top two kinases (MAPK1 and AKT1) are reported in these patients. MAPK1 and AKT1 showed variable expression among the patients (Figure 6B). 25% and 31% of these individuals had altered MAPK1 and AKT1 expression with a z-score cut off of 1(Supplementary Figure S2).

We further checked the significant difference in expression of the top two predicted kinases in mRNA as well as protein data. We observed, of the 30% of the patients with altered mRNA expression of MAPK1, 21 patients showed high expression and 18 patients showed lower expression (z-score>1) with significant difference in expression with in the patient cohort (*p*-value = 9.73e^−15^). Similarly, 22 patients exhibited higher expression of AKT1 and eight patients showed lower expression for AKT1 mRNA expression (z-score>1). The AKT1 showed significant difference in mRNA expression among the altered cohort (Figure 6C). Similarly, we checked the protein expression of MAPK1 and AKT1, eight patients each showed high expression of both the proteins whereas 7 and 11 patients respectively showed lower expression of MAPK1 and AKT1 (Figure 6D).

### 3.7 Phosphoproteomics of NACT resistant patients revealed several druggable targets

We sought to identify the druggable targets that were enriched in our data. We searched the hyperphosphorylated kinases and predicted kinases in the Therapeutic Target Database (TTD) and we found many of the inhibitors for the enriched kinases are either approved or in the clinical trial (Table 1). For example, ravoxertinib and ulixertinib which inhibit MAPK1 and 3, are currently under clinical trial. In our data, MAPK3 is predicted to be enriched in the non-responders.

**TABLE 1:** Kinases and the clinical trial status of corresponding inhibitors along with current application in other cancers.

Kinases	Inhibitors	Clinical trial status	Drug’s application in other cancers
FGFR3	Pemigatinib	Approved	Cholangiocarcinoma
	Vofatamab	Phase1	Multiple myeloma
	E-7090	Preclinical	Breast carcinoma
	LY-2874455	Preclinical	Advanced solid tumors
	BMS-582664	Phase III	Hepatocellular carcinoma
	E-3810	Phase III	Solid tumour/cancer
SLK	Danusertib		Ovarian carcinoma
	Hesperadin		
RSK1_b	Prexasertib	Phase I/II	mCRPC, Leukemia, Breast and Ovarian carcinoma
	Dorsomorphin		
	BVD-523	Phase II	Melanoma
	ASTX029	Phase I/II	Solid tumor
	Ravoxertinib	Under clinical trial	Locally advanced/metastatic solid tumor
	Ulixertinib	EAP granted	MAPK pathway aberrant cancers
PRKCZ	Methyl 2-amino-4-phenylthiophene-3-carboxylate		Solid tumors
MAPK1	Ravoxertinib	Under clinical trial EAP granted	Locally advanced/metastatic solid tumo
	Ulixertinib	FDA approved	MAPK pathway aberrant cancers,
	BVD-523	Phase II	Melanoma
	ASTX029	Phase 1/2	Solid tumour/cancer
MAPK7	Sorafenib	FDA Approval	Hepatocellular carcinoma, Renal cell carcinoma, Thyroid carcinoma
AKT1	Ipatasertib	Phase II	Triple negative breast cancer
	Capivasertib	Phase I	Ovarian cancer, Melanoma
	MK-2206+Vemurafenib		
	(Combination therapy)		
	AZD5363	Phase III	Triple negative breast cancer
	Enzastaurin	Phase III	Non-hodgkin lymphoma

## 4 Discussion

Tongue squamous cell carcinoma (TSCC) remains the most common oral cavity cancer with a significant incidence as well as mortality. Treatment strategies such as surgical resections along with modern reconstructive methods and adjuvant therapies could improve the locoregional control of the disease. However, the overall survival of oral tongue cancer has not improved in the past decades (Chinn and Myers, 2015). Although neoadjuvant chemotherapy is regarded for better clinical outcome and organ preservation, the resistance to the treatment limits the clinical outcome. In this study, we employed mass spectrometry-based global proteomics as well as phosphoproteomics of treatment-naive biopsy samples from TSCC patients to identify the differentially expressed proteins, kinases and elucidated the mechanisms associated with neoadjuvant chemotherapy resistance.

Abnormal expression of keratins is reported in cancer cells and the keratin profiling of oral squamous cell carcinoma exhibited higher expression of keratins such as KRT6, KRT16, and KRT17 (Moll et al., 2008; Sakamoto et al., 2011). Our proteomic data revealed higher expression type II keratin, KRT1, and its heterodimer type I partner KRT10 along with other keratins such as KRT72, KRT71, and KRT84. Previous studies have linked altered KRT10 expression to the dysplastic progression of oral cancer (Ali et al., 2018). In addition, expression of Keratin 1/10 was identified in premalignant oral lesions (Kannan et al., 1994; Ali et al., 2018). Interestingly, we also observed high and extensive phosphorylation of type I (KRT) and type II (KRT) keratins. Phosphorylation of keratins regulates the physical properties of the cancer cells and persistent phosphorylation of keratins can cause disintegration of keratins which leads to weaker adhesion of cells, increased migration, invasion and is associated with metastatic cancer (Kim et al., 2015). The differential phosphorylation status of keratins between the cohort might be a contributing factor for treatment resistance.

Further, our pathway analysis of phosphoproteomic data illustrated enrichment of the Rho GTPase cycle and signaling by Rho GTPase pathway. Rho GTPase, the classical drivers of cytoskeletal dynamics and migration, which also known to involve in cell survival, and tumor progression (Haga and Ridley, 2016). We observed differential phosphorylation of proteins involved in Rho signaling in non-responders. For example, TRIP10 downstream effector of CDC42 is hyperphosphorylated in non-responder cohorts and in triple negative breast cancer, the hypermethylation of TRIP10 is related with the prediction of response to neoadjuvant chemotherapy (Pineda et al., 2019). Rho guanine nucleotide exchange factors (GEFs) (RASGRF2, RAPGEF1, ARHGEF6, DOCK2) and GTPase-activating proteins (GAPs) (ARFGAP2, ARHGAP30) were also differentially phosphorylated. Phosphorylation of Ras Protein Specific Guanine Nucleotide Releasing Factor 2 (RASGRF2) inhibits the active Rac and there by ERK 1, two pathways (Kesavapany et al., 2004). RASGRF2 is hypophosphorylated in non-responders, moreover, the kinase-substrate enrichment analysis revealed the activation of kinases such as ERK1 and ERK 2. Rho and Raf/MEK/ERK signalling pathways are cross-linked and play a role in oncogenic transformation of cells (Soriano et al., 2021). This suggests activation of Rho signaling might have further activated MAPK in non-responders.

**FIGURE 7:**
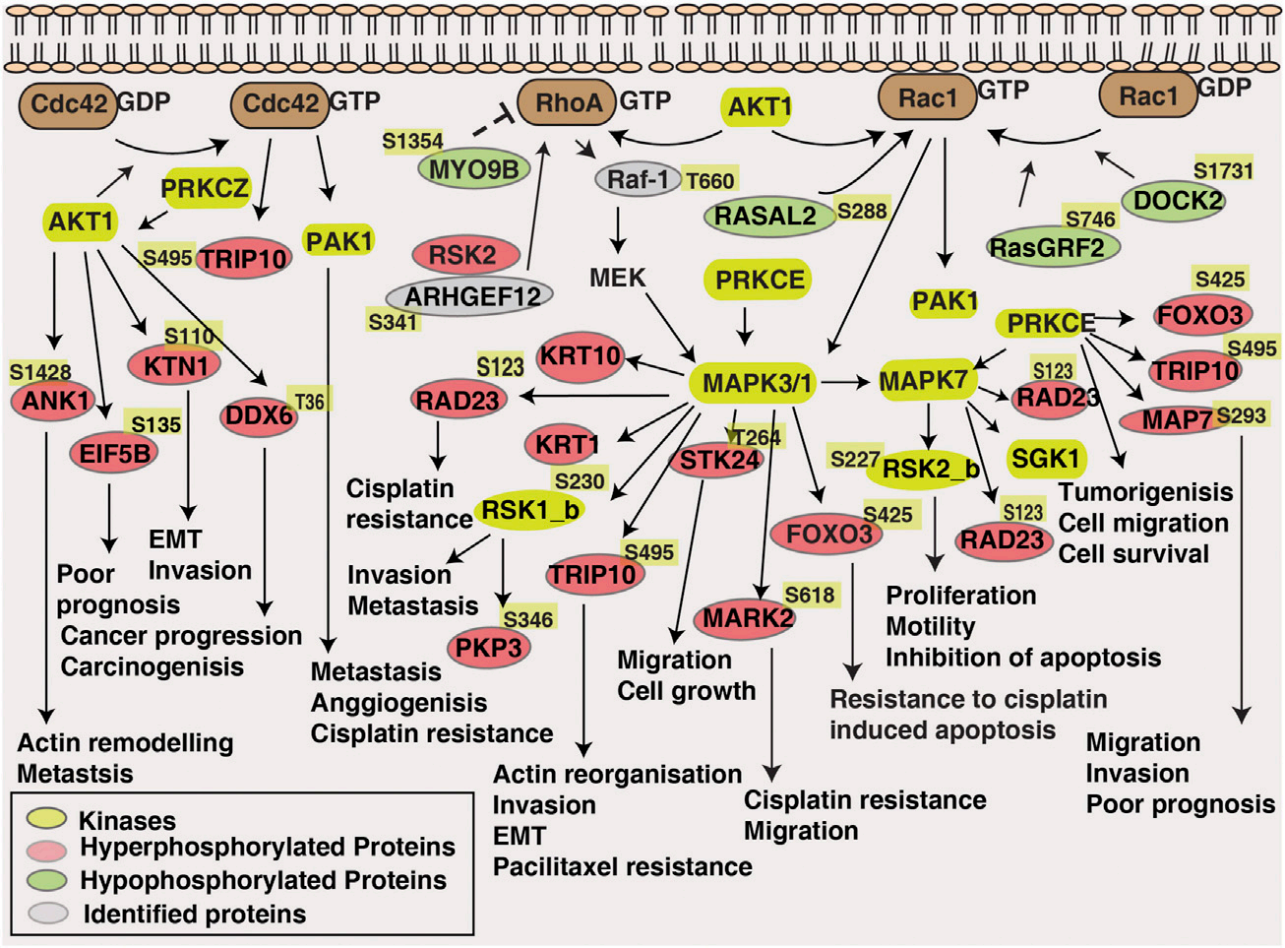
Data-driven model of NACT resistance in tongue cancer patients. Pathway analysis revealed the enrichment of Rho-GTPase signalling pathway in NACT resistant patients. Dysregulated proteins, differentially phosphorylated kinases and proteins involved in Rho-GTPase pathway is highlighted in the schematic diagram.

Kinases such as MAPK1, AKT, MAPK3 and MAPK7 were predicted to be enriched in non-responders and several downstream substrates of these kinases were also hyperphosphorylated in the non-responder cohort. For example, FOXO3, downstream substrate of ERK, SGK and AKT was hyperphosphorylated at the site Ser425 in non-responders. Phosphorylation of Ser425 of FOXO3 by ERK leads to its inactivation and further activates cell proliferation and resistance to apoptosis (Yang et al., 2008). Kinases such as PRKCZ and PRKCE, which are the positively regulated by ERK1,2,5 cascade were also enriched in the non-responder cohort. Microtubule associated protein 7 (MAP7), the downstream substrate of PRKCE was hyperphosphorylated in non-responder cohort and the influence of MAPs in microtubule targeting drugs such as targeting drugs such as paclitaxel and docetaxel is previously reported (Fromes et al., 1996). Downstream kinases of MAPKs such as MARK2, RSK1_b, RSK2_b, were also hyperphosphorylated in non-responders and the role of these kinases in drug resistance is previously reported in independent studies (van Jaarsveld et al., 2013; Hubaux et al., 2015; Salhi et al., 2015). Also, activity of kinases such as SRC and ABL1 were predicted to be downregulated in non-responders. Activation of the upstream phosphatases might be one of the reasons for the downregulation of these kinases. PTPRA, an upstream phosphatase of SRC is reported for its oncogenic role and is highly expressed in head and neck cancers (Julien et al., 2011). High expression of PTPRA led to the dephosphorylation of SRC and thereby FAK mediated cell migration in epidermoid carcinoma cells (Harder et al., 1998). For confirming these primary observations and to elucidate the impact of the kinases and phosphatases in treatment response of tongue cancer further validation studies have to be carried out. Overall, our data suggests hyperphosphorylation of the substrates in non-responders by the MAPKs and AKT might be conferring NACT resistance. Figure 7 represents a data driven model of mechanism of neoadjuvant chemotherapy resistance in tongue cancer patients.

Our findings imply that differential MAPK and AKT expression patterns may be causing varied NACT responses in tongue cancer. We therefore sought to check the expression of predicted kinases in the publicly available tongue cancer data bases. Alteration of MAPK1 and AKT1 were found in the mRNA data (TCGA), and this was also observed in the protein data base (RPPA). In concordance with our data, we observed significant difference in expression of these kinases across the tongue cancer patients. In a study on triple negative breast cancer, it has been shown the positive expression of MAPK1 with tumor recurrence and poor overall survival (Jiang et al., 2020). Due to the lack of data on NACT response on tongue cancer in public databases, it limits to draw conclusive correlation on MAPK1 and AKT1 expression with treatment resistance. Further mechanistic studies are required to elucidate the role of MAPK1 and AKT1 in treatment resistance of tongue cancer.

Kinase inhibitors has proven to improve the clinical efficacy compared to the conventional therapy procedures. Food and Drug Administration (FDA) has approved multiple kinase inhibitors and several inhibitors are in different trial stages. It is noticeable that in non-responder cohort activation of MAPKs and AKT cohort further activated several downstream kinases, suggesting MAPKs and AKT could be potential targets in these patients. However, the association of these activated kinases with the clinical outcome should be further validated.

Our results suggest extensive phosphorylation of cytoskeleton component; keratins and activation of Rho GTPase signalling and kinases might have facilitated differential treatment response between the two patient cohorts. Activation of MAPKs and AKT kinase and the differentially phosphorylated proteins might have further contributed to treatment resistance in non-responder cohort. Although, our results provide information about the key players in NACT resistance of tongue cancer, multicentric validation studies have to be carried out in larger cohort to assure the reproducibility of the findings.

## 5 Conclusion

Our findings reveal a clear difference in proteomic and phosphoproteomic signatures between the responder and non-responder tongue cancer patients. The altered phosphorylation of proteins, activated kinases and more over the activation of Rho GTPase signalling pathways promote therapy resistance in non-responders. Our results suggest MAPK1, AKT1, and MAPK3 as potential drug targets in NACT resistant tongue cancer patients. However, further validation studies should be carried out to consolidate our findings.

## Data Availability

The datasets are available via ProteomeXchange with identifier PXD037356 (http://www.proteomexchange.org).
