# Supplementary material for: Integrated proteomics and phosphoproteomics revealed druggable kinases in neoadjuvant chemotherapy resistant tongue cancer
Source: Front Cell Dev Biol. 2022 Oct 28;10:957983. doi: 10.3389/fcell.2022.957983 (PMC9651967; doi:10.3389/fcell.2022.957983)
Supplement: Supplementary file 2 [file DataSheet1.docx]

**Supplementary figures**

**Manuscript Id:** 957983

**Manuscript Title: Integrated proteomics and phosphoproteomics revealed druggable kinases in neoadjuvant chemotherapy resistant tongue cancer**

**Supplementary figure 1:** Protein prtein interaction network for the 81 proteins which are dysregulated at phosphorylation level without any change in protein expression. Phsophosites highligheted in red are hyperphosphorylated proteins and phsophosite highlighetd in green are hypophosphorylated. Interaction score=0.70; proteins which have no interactions are removed from the analysis.


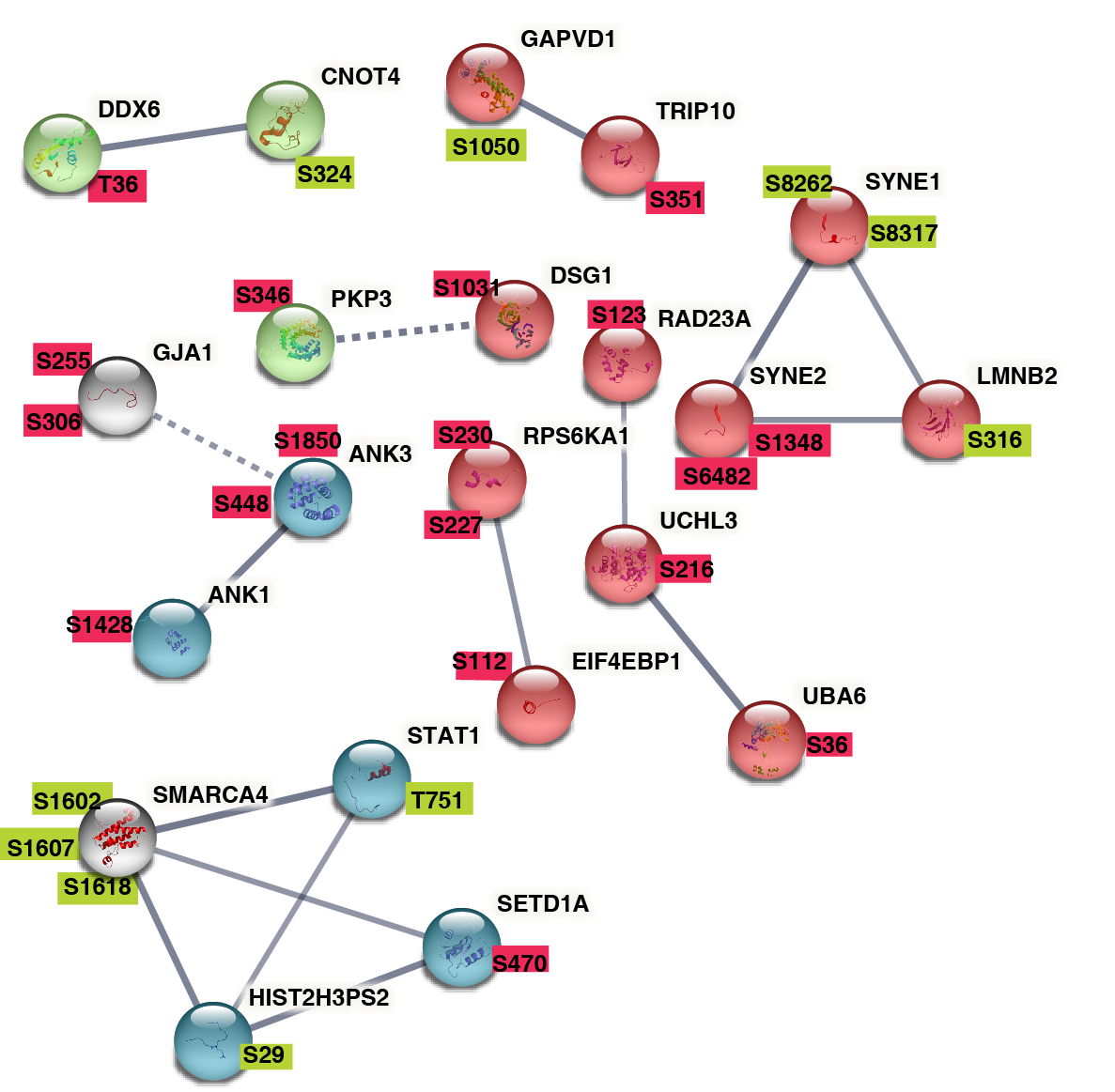


Number of nodes= 81; number of edges=17; average nodes degree=0.42 PPI erichment p-value=0.0387

**Supplementary figure 2**: MeanRank visualization from KEA3 for the down-phosphorylated proteins.


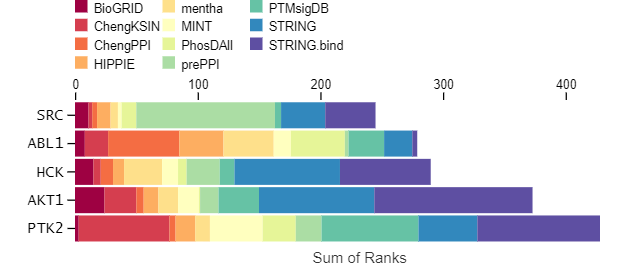


**Supplementary figure 3**: Oncoprint showing alteration in mRNA expression of the top ten predicted kinases.

| 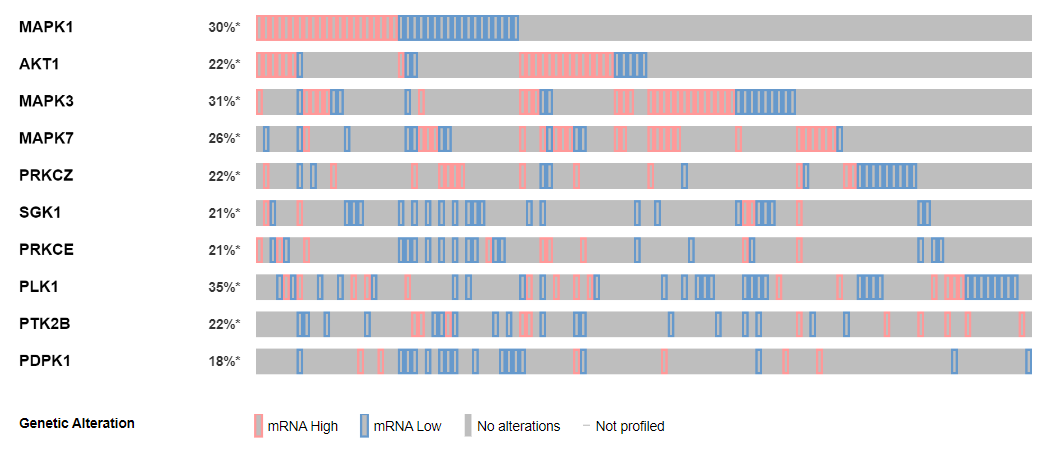 |
| --- |
|  |
|  |
|  |
|  |
|  |
| **Supplementary figure 4**: Oncoprint showing the alteration in protein expression of top ten predicted kinases. |
|  |
| 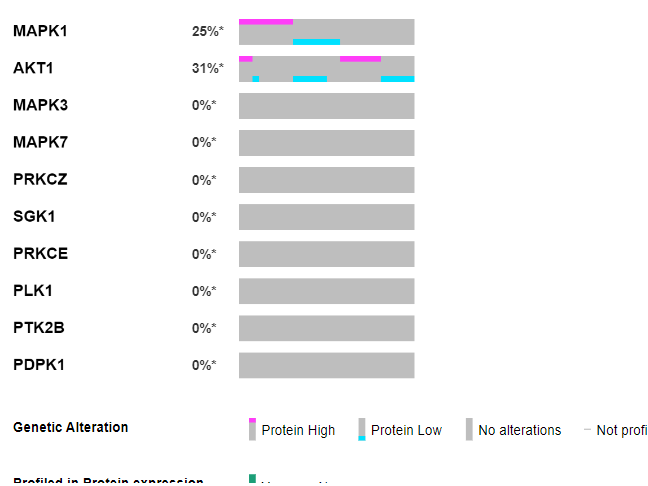 |
|  |
|  |
|  |
